# N‐terminal alterations turn the gut hormone GLP‐2 into an antagonist with gradual loss of GLP‐2 receptor selectivity towards more GLP‐1 receptor interaction

**DOI:** 10.1111/bph.15866

**Published:** 2022-06-08

**Authors:** Maria Buur Nordskov Gabe, Lærke Smidt Gasbjerg, Sarina Gadgaard, Peter Lindquist, Jens Juul Holst, Mette Marie Rosenkilde

**Affiliations:** ^1^ Department of Biomedical Sciences, Faculty of Health and Medical Sciences University of Copenhagen Copenhagen Denmark; ^2^ Novo Nordisk Foundation Center for Basic Metabolic Research, Faculty of Health and Medical Sciences University of Copenhagen Copenhagen Denmark

**Keywords:** antagonists, family B1 GPCR, GLP‐1 receptor, GLP‐2, GLP‐2 receptor, GPCR, N‐terminus

## Abstract

**Background and Purpose:**

To fully elucidate the regulatory role of the GLP‐2 system in the gut and the bones, potent and selective GLP‐2 receptor (GLP‐2R) antagonists are needed. Searching for antagonist activity, we performed systematic N‐terminal truncations of human GLP‐2(1‐33).

**Experimental Approach:**

COS‐7 cells were transfected with the human GLP‐2R and assessed for cAMP accumulation or competition binding using ^125^I‐GLP‐2(1‐33)[M10Y]. To examine selectivity, COS‐7 cells expressing human GLP‐1 or GIP receptors were assessed for cAMP accumulation.

**Key Results:**

Affinity of the N‐terminally truncated GLP‐2 peptides for the GLP‐2 receptor decreased with reduced N‐terminal peptide length (K_i_ 6.5–871 nM), while increasing antagonism appeared with inhibitory potencies (IC_50_) values from 79 to 204 nM for truncation up to GLP‐2(4‐33) and then declined. In contrast, truncation‐dependent increases in intrinsic activity were observed from an E_max_ of only 20% for GLP‐(2‐33) up to 46% for GLP‐2(6‐33) at 1 μM, followed by a decline. GLP‐2(9‐33) had the highest intrinsic efficacy (E_max_ 65%) and no antagonistic properties. Moreover, with truncations up to GLP‐2(8‐33), a gradual loss in selectivity for the GLP‐2 receptor appeared with increasing GLP‐1 receptor (GLP‐1R) inhibition (up to 73% at 1 μM). Lipidation of the peptides improved antagonism (IC_50_ down to 7.9 nM) for both the GLP‐2 and the GLP‐1R.

**Conclusion and Implications:**

The N‐terminus of GLP‐2 is crucial for GLP‐2R activity and selectivity. Our observations form the basis for the development of tool compounds for further characterization of the GLP‐2 system.

**Abbreviations**
BHK, baby hamster kidney; BSA: bovine serum albumin
COS‐7: african green monkey kidney fibroblast‐like
DPP‐4: dipeptidyl peptidase‐4
cAMP: 3'‐5'‐cyclic adenosine monophosphate
GIP: glucose‐dependent insulinotropic polypeptide
GIPR: GIP receptor
GLP‐1: glucagon‐like peptide‐1
GLP‐1R: GLP‐1 receptor
GLP‐2: glucagon‐like peptide‐2
GLP‐2R: GLP‐2 receptor
GPCR: G protein‐coupled receptor
HBS: HEPES buffered saline
HEK: human embryonic kidney
SBS: short bowel syndrome

**What is already known**

- GLP‐2(3‐33) is a partial agonist at GLP‐2 receptor with antagonistic actions in vivo.
- Potent and selective GLP‐2 receptor antagonists allowing better characterization of the GLP‐2 system are needed.

**What does this study add**

- N‐terminally truncated GLP‐2 peptides act as antagonists at the GLP‐2 receptor.
- Selectivity for the GLP‐2 receptor over the GLP‐1 receptor decreases with reduced N‐terminal GLP‐2 peptide length.

**What is the clinical significance**

- Our results can form the basis for development of selective GLP‐2‐based tool compounds.

## 1 INTRODUCTION

Glucagon‐like peptide‐2 (GLP‐2) is a 33‐amino acid intestinal hormone primarily known for its roles in the regulation of intestinal mucosal growth, gastrointestinal motility and bone metabolism (Drucker & Yusta, 2014; Hartmann et al., 2000; Jeppesen, 2006; Skov‐Jeppesen et al., 2019). The GLP‐2 analogue, teduglutide, is resistant to cleavage by dipeptidyl peptidase‐4 (DPP‐4) and is used in the treatment of patients with short bowel syndrome (SBS), where it results in reduced gastric emptying and stomal output, higher intestinal energy absorption and weight gain (Bremholm et al., 2009; Drucker & Yusta, 2014; Jeppesen et al., 2001), sometimes enabling the patients to avoid parenteral nutrition. Furthermore, GLP‐2 influences bone remodelling, and exogenous administration of the peptide decreased bone resorption (Askov‐Hansen et al., 2013; Gottschalck et al., 2008; Henriksen et al., 2003, 2009; Skov‐Jeppesen et al., 2019). In addition, in rodents fed with high fat diets, GLP‐2 increased hepatic lipogenesis (Taher et al., 2018), insulin sensitivity and glucose tolerance (Baldassano et al., 2016) and, furthermore, inhibited hepatic glucose production (Shi et al., 2013). The naturally occurring DPP‐4 cleavage product of GLP‐2, GLP‐2(3‐33), has previously been shown to be a low potency, partial agonist of the GLP‐2R both in vitro and in vivo in rodents (Thulesen et al., 2002; Yamazaki et al., 2013) but no potent GLP‐2R antagonists have at present been described.

For another class B1 receptor, the GLP‐2R‐related glucagon‐like peptide‐1 (GLP‐1) receptor (GLP‐1R), the identification and use of the antagonist, exendin(9–39)NH_2_ , has been an essential tool for exploration of the GLP‐1 system both in vitro and, more importantly, in human studies (Edwards et al., 1999; Gasbjerg et al., 2021; Jørgensen et al., 2013; Nicolaus et al., 2011; Raufman et al., 1991; Sathananthan et al., 2013; Schirra et al., 2009; Thorens et al., 1993). This has led to detailed characterization of the involvement of GLP‐1 in physiological and pathophysiological mechanisms which underlie the use of GLP‐1R agonists as anti‐diabetic and anti‐obesity agents (Andersen et al., 2018). Inspired by this, an effective and selective antagonist of the likewise closely related class B1 receptor of the glucose‐dependent insulinotropic polypeptide (GIP) was recently identified in the form of GIP(3–30)NH_2_ —the natural occurring DPP‐4 degradation product of GIP(1–30)NH_2_ (Gabe et al., 2018; Hansen et al., 2016; Sparre‐Ulrich et al., 2017). This GIP receptor (GIPR) antagonist has enabled a multitude of in vitro and clinical studies allowing detailed characterization of the GIP system (Gasbjerg et al., 2017, 2018, 2019, 2020; Skov‐Jeppesen et al., 2019). Because the physiological relevance of many of the actions described for GLP‐2 and the knowledge about the actions of endogenous GLP‐2 are uncertain, availability of a GLP‐2R antagonist is similarly important. In addition to this, a GLP‐2R antagonist could have a therapeutic potential in the long run; however, a careful characterization of the physiology of the GLP‐2 system is foremost needed.

To be able to design potent GLP‐2R antagonists, a thorough understanding of the receptor activation mechanism is important. As for both the GIPR (Smit et al., 2021; Zhao et al., 2021) and GLP‐1R (Schwartz & Frimurer, 2017), a cryo‐EM structure of the GLP‐2R was recently published (Sun et al., 2020). Here the importance of the GLP‐2 N‐terminus for GLP‐2R activation was confirmed as both the N‐terminal His at position 1 and the Asp at position 3 form hydrogen bonds and hydrophobic contacts with several amino acids of the GLP‐2R (Sun et al., 2020). Thus, removing residues of the N‐terminus from the GLP‐2 peptide would theoretically be a reasonable approach in the design of GLP‐2R antagonists.

In a systematic approach, we here present how the binding and activation profiles of the human GLP‐2R are affected by 10 N‐terminally truncated GLP‐2 peptides, and whether this resulted in development of antagonists for the human GLP‐2R. To examine whether the specificity of GLP‐2 lies in the N‐terminus of the peptide, we also studied the selectivity of the truncated variants with respect to the closely related human GLP‐1R and GIPR. With a view to improve the usefulness of the GLP‐2R antagonists identified and make them suitable for in vivo studies, we optimized their pharmacokinetic profiles by site‐specific lipidation.

## 2 METHODS

### 2.1 Transfection and tissue cultures

COS‐7 cells (RRID:CVCL_0224) were cultured at 10% CO_2_ and 37°C in Dulbecco's modified Eagle's medium (DMEM) 1885 supplemented with 10% fetal bovine serum (FBS), 2 mM glutamine, 180 units·ml^−1^ penicillin and 45 g·ml^−1^ streptomycin. Transient transfection of COS‐7 cells was performed using the calcium phosphate precipitation method as previously described (van der Velden et al., 2021).

### 2.2 Heterologous competition binding

COS‐7 cells transiently expressing the human GLP‐2R were seeded in clear 24‐well plates 1 day after transfection at a density of 100,000 cells per well. The number of cells per well was chosen to achieve 5%–10% specific binding of the radioligand, [^125^I]‐GLP‐2(1‐33)[M10Y]. The following day, the cells were assayed by competition binding for 3 h at 4°C using 15–40 pM of [^125^I]‐GLP‐2(1‐33)[M10Y] as well as unlabelled ligand in a total volume of 210 μl per well in 50 mM HEPES buffer (pH 7.2) supplemented with 0.5% bovine serum albumin (BSA) (binding buffer). After incubation, the cells were washed twice in 400 μl per well ice‐cold binding buffer and lysed using 500 μl per well of 200 mM NaOH with 1% SDS for 30 min. The samples were counted using the Wizard Gamma Counter (PerkinElmer, Waltham, MA).

### 2.3 cAMP assay

Transiently transfected COS‐7 cells were seeded in white 96‐well plates the day after transfection at a density of 35,000 cells per well. The next day, the assay was initiated by washing with HEPES buffered saline (HBS), followed by an incubation step with assay buffer (HBS containing 1 mM 3‐isobutyl‐1‐methylxanthine [IBMX]) for 30 min at 37°C. Before addition, the assay buffer was adjusted to pH = 8.3 using a 4 M NaOH stock. To test for agonism, the ligands were added and incubated for an additional 30 min at 37°C. To test for antagonistic properties, the cells were preincubated for 10 min with the antagonist with subsequent addition of the agonist and incubation for an additional 20 min. After ligand incubation, the HitHunter® cAMP assay (Eurofins DiscoverX, Fremont, USA) was carried out according to the manufacturer's instructions. Luminescence was measured by PerkinElmer™ EnVision 2014 Multilabel Reader (PerkinElmer, Waltham, MA).

### 2.4 Data and statistical analysis

IC_50_, EC_50_ and K_i_ values were determined by non‐linear regression using GraphPad Prism 9 (San Diego, California, USA) (GraphPad Prism, RRID:SCR_002798). Sigmoid curves were fitted logistically with a Hill slope of 1.0 for the cAMP activation curves and −1.0 for the inhibition of cAMP and binding. K_i_ values were calculated using the Cheng–Prusoff formula under the assumption of one class of binding sites. Dose ratios (DR) for the Schild analyses were calculated from the potency shift of GLP‐2 in the presence of a given GLP‐2R antagonist concentration, relative to that in the the absence of GLP‐2R antagonist. In order to use equieffective DR for experiments including the partial agonists, the concentrations causing 60% of E_max_ were applied enabling a valid Schild analysis. Schild plots were made with log(DR‐1) (ordinate) and log(antagonist concentration) (abscissa) to estimate the slopes and pA_2_ values. To decease unwanted variations of the assay outputs (cAMP assays), each experiment, performed in duplicate, was normalized to the absence of ligand (i.e., only buffer addition) as baseline (0%) and the highest tested concentration of the endogenous hormone (100%). Statistical analysis was undertaken only for studies where each group size was at least *n* = 5 and only if relevant. The declared group size is the number of independent values and the statistical analysis was done using these independent values. Statistical significance was accepted at *P* < 0.05. Outliers were in general included in the data analysis and presentation except for one assay in the Schild plot analysis of GLP‐2(2‐33), which behaved completely opposite of all the five other assays. All in vitro experiments were repeated at least three times, in duplicate, and if a large variation between experiments were observed, additional experiments were included. The manuscript complies with *BJP*'s recommendations and requirements on experimental design and analysis (Curtis et al., 2018).

### 2.5 Materials

Human GLP‐2, GLP‐1 and GIP were purchased from Bachem, Bubendorf, Switzerland (4039611, 4030663 and 4030658, respectively). The N‐terminally truncated GLP‐2 peptides were synthesized by CASLO ApS, Lyngby, Denmark. All peptides had a purity of at least 95% by HPLC analysis and correct mass spectrometry‐controlled molecular weight. cDNAs of human GLP‐2R, human GLP‐1R and human GIPR were purchased from Origene, Rockville, Maryland, USA (SC111108, SC124060 and SC110906, respectively). GLP‐2(1‐33)[M10Y] was [^125^I]‐labelled using the standard stoichiometric chloramine T method (Gadgaard et al., 2021).

### 2.6 Nomenclature of targets and ligands

Key protein targets and ligands in this article are hyperlinked to corresponding entries in http://www.guidetopharmacology.org and are permanently archived in the Concise Guide to PHARMACOLOGY 2021/22 (Alexander, Christopoulos et al., 2021; Alexander, Fabbro et al., 2021).

## 3 RESULTS

**FIGURE 1:**
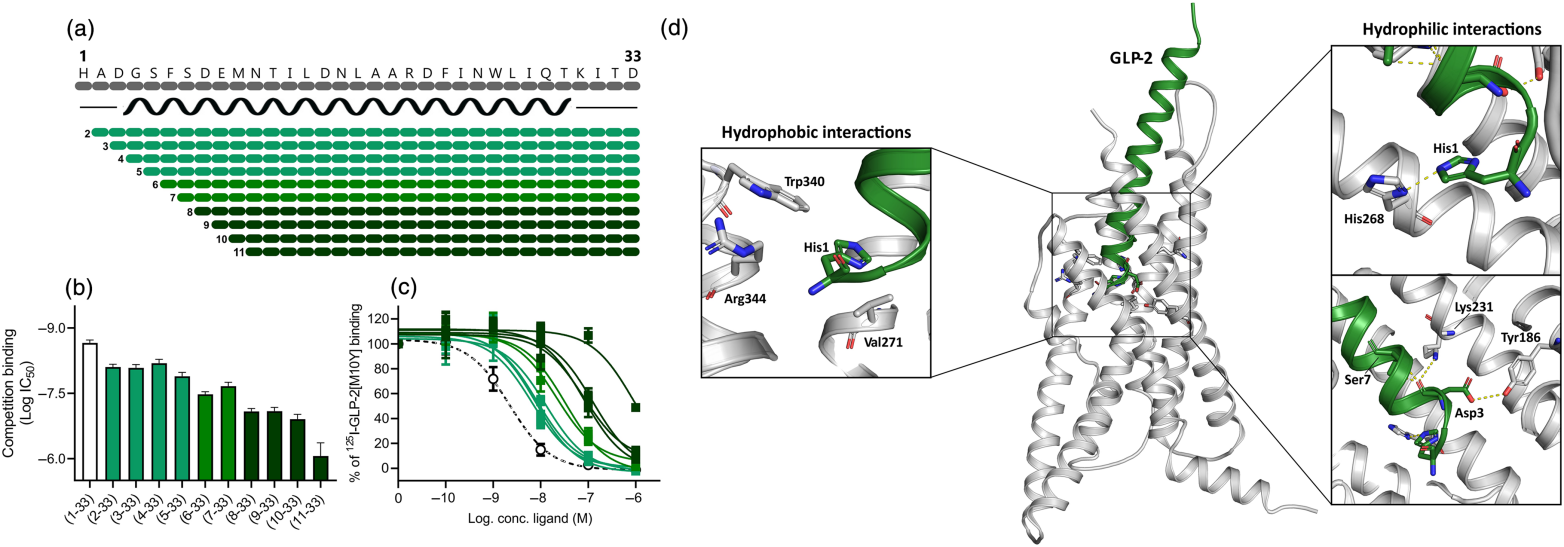
N‐terminally truncated GLP‐2 variants on the human GLP‐2R. (a) Schematic overview of the N‐terminally truncated GLP‐2 variants. The black spiral indicates the predicted α‐helix structure from amino acid number 4 to amino acid number 29 (34). COS‐7 cells were transiently transfected with the human GLP‐2R and the N‐terminally truncated GLP‐2 variants competed with [^125^I]‐GLP‐2(1‐33)[M10Y] giving the (b) log IC_50_ values calculated from (c) the inhibition curves. The dashed line in (c) represents human GLP‐2. Data are shown as mean ± SEM, from *n* = 3 independent experiments carried out in duplicate. (d) A representation of the human GLP‐2R structure (grey—PDBid: 7D68) in complex with the full length GLP‐2 peptide (green) is showed where the interactions between the N‐terminal GLP‐2 residues and the GLP‐2R are highlighted.

The N‐terminal truncations were made in the sequence of human GLP‐2 where up to 10 amino acids were removed (Figure 1a). In vitro, a detailed characterization of the binding and cAMP activation profiles of the N‐terminal GLP‐2 truncations was performed.

### 3.1 Differential binding profiles of N‐terminally truncated GLP‐2 peptides

We studied the role of the GLP‐2 N‐terminus in binding to the GLP‐2R, by the ability of the truncated GLP‐2 peptides to compete with [^125^I]‐GLP‐2(1‐33)[M10Y] (Gadgaard et al., 2021). Here we found that GLP‐2(2‐33), GLP‐2(3‐33), GLP‐2(4‐33) and GLP‐2(5‐33) had <10‐fold decreased affinities compared with native GLP‐2 (3.6‐, 3.7‐, 3.0‐ and 5.9‐fold lower affinity, respectively) (Figure 1b/c). GLP‐2(6‐33) and GLP‐2(7‐33) had a 15‐ and 10‐fold impaired affinity compared with native GLP‐2, whereas a 37‐fold impaired affinity was observed for both GLP‐2(8‐33) and GLP‐2(9‐33) and a 56‐fold impaired affinity for GLP‐2(10‐33) (Figure 1b/c). GLP‐2(11‐33) had a >350‐fold shift in its affinity compared with native GLP‐2 (Figure 1b/c). Thus, the affinity of the N‐terminal truncations became gradually weaker for increasing N‐terminal truncations.

### 3.2 N‐terminal truncations of GLP‐2 result in antagonists of the GLP‐2R

**FIGURE 2:**
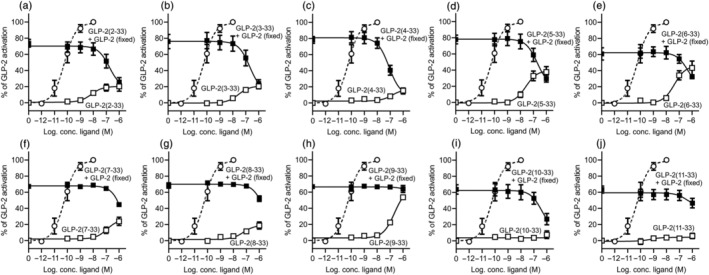
cAMP activation and inhibition profiles of N‐terminally truncated GLP‐2 variants on the human GLP‐2R. COS‐7 cells were transiently transfected with human GLP‐2R and assessed for either cAMP activation or inhibition upon stimulation with the GLP‐2 truncated variants of (a) GLP‐2(2‐33), (b) GLP‐2(3‐33), (c) GLP‐2(4‐33), (d) GLP‐2(5‐33), (e) GLP‐2(6‐33), (f) GLP‐2(7‐33), (g) GLP‐2(8‐33), (h) GLP‐2(9‐33), (i) GLP‐2(10‐33) or (j) GLP‐2(11‐33). The dashed line in each graph represents human GLP‐2. Data are shown as mean ± SEM, from *n* = 3 independent experiments carried out in duplicate

As Gα_s_ coupling is the main signalling pathway for the GLP‐2R (Yusta et al., 1999), we went on to study the activity profiles of the peptides in terms of their ability to activate and inhibit this receptor with respect to cAMP accumulation (Figure 2). As the N‐terminus plays a major role in GLP‐2R activation (Sun et al., 2020), we expected that removing amino acids from the N‐terminus would, most likely, generate peptides with an impaired activation profile. Compared with GLP‐2(1‐33), GLP‐2(2‐ to 4‐33) activated the GLP‐2R with very low efficacy (Figure 2a–c); however, surprisingly, GLP‐2(5‐ to 6‐33) resulted in peptides that activated the GLP‐2R with increasing efficacy (Figure 2d,e, Table 1). The subsequent truncations, GLP‐2(7‐ to 11‐33), activated the GLP‐2R with very low efficacy or not at all except for GLP‐2(9‐33), which showed low potent partial agonistic activity with an efficacy of 65% at 1 μM stimulation (Figure 2f–j, Table 1). When looking at the antagonistic properties of the N‐terminal GLP‐2 truncations, GLP‐2(2‐33), GLP‐2(3‐33), GLP‐2(4‐33) and GLP‐2(5‐33) had the strongest antagonistic properties with IC_50_ values ranging between 79 and 204 nM where GLP‐2(4‐33) displayed the most pronounced inhibition (Figure 2a–d, Table 1). GLP‐2(6‐ to 8‐33) showed <50% inhibition at 1 μM (Figure 2e–g, Table 1), GLP‐2(9‐33) and GLP‐2(11‐33) were not able to antagonize the GLP‐2‐induced activation of the GLP‐2R (Figure 2h,j) whereas GLP‐2(10‐33) only weakly inhibited the activation (Figure 2i).

**TABLE 1:** Affinity, potency and efficacy values of the N‐terminal truncated GLP‐2 variants on the GLP‐2R

	Competition binding		cAMP activation			cAMP inhibition	
	Log IC_50_ ± SEM	K_i_ (nM)	Log EC_50_ ± SEM	EC_50_ (nM)	E_max_ ± SEM (%)	Log IC_50_ ± SEM	IC_50_ (nM)
GLP‐2(1‐33)	−8.7 ± 0.12	2.2	−10.2 ± 0.14	0.06	100 ± 4.7	‐	‐
GLP‐2(2‐33)	−8.1 ± 0.11	7.9	−8.1 ± 0.50	7.9	20 ± 3.2	−6.8 ± 0.34	174
GLP‐2(3‐33)	−8.1 ± 0.13	8.1	−7.6 ± 0.26	25	21 ± 2.2	−6.7 ± 0.39	204
GLP‐2(4‐33)	−8.2 ± 0.16	6.5	−6.8 ± 0.30	158	17 ± 2.6	−7.1 ± 0.18	79
GLP‐2(5‐33)	−7.9 ± 0.15	12	−7.6 ± 0.24	25	40 ± 4.1	−6.7 ± 0.34	200
GLP‐2(6‐33)	−7.5 ± 0.11	33	−7.4 ± 0.24	40	46 ± 5.1	−6.3 ± 0.71	490
GLP‐2(7‐33)	−7.7 ± 0.15	22	−6.9 ± 0.24	126	27 ± 3.8	No inhibition	
GLP‐2(8‐33)	−7.1 ± 0.12	81	−7.2 ± 0.31	63	19 ± 3.0	No inhibition	
GLP‐2(9‐33)	−7.1 ± 0.15	81	−6.7 ± 0.08	200	65 ± 3.6	No inhibition	
GLP‐2(10‐33)	−6.9 ± 0.19	123	No activation			−6.39 ± 0.66	398
GLP‐2(11‐33)	−6.1 ± 0.53	871	No activation			No inhibition	

*Note*: The table displays a summary of the affinity, potency and efficacy values of the N‐terminal truncated GLP‐2 variants on the human GLP‐2R tested in the binding and cAMP accumulation experiments. The values originate from the data shown in Figures 1 and 2 from *n* = 3 independent experiments carried out in duplicate.

### 3.3 GLP‐2(3‐33)[D3A] is a GLP‐2R antagonist with no intrinsic activity

**FIGURE 3:**
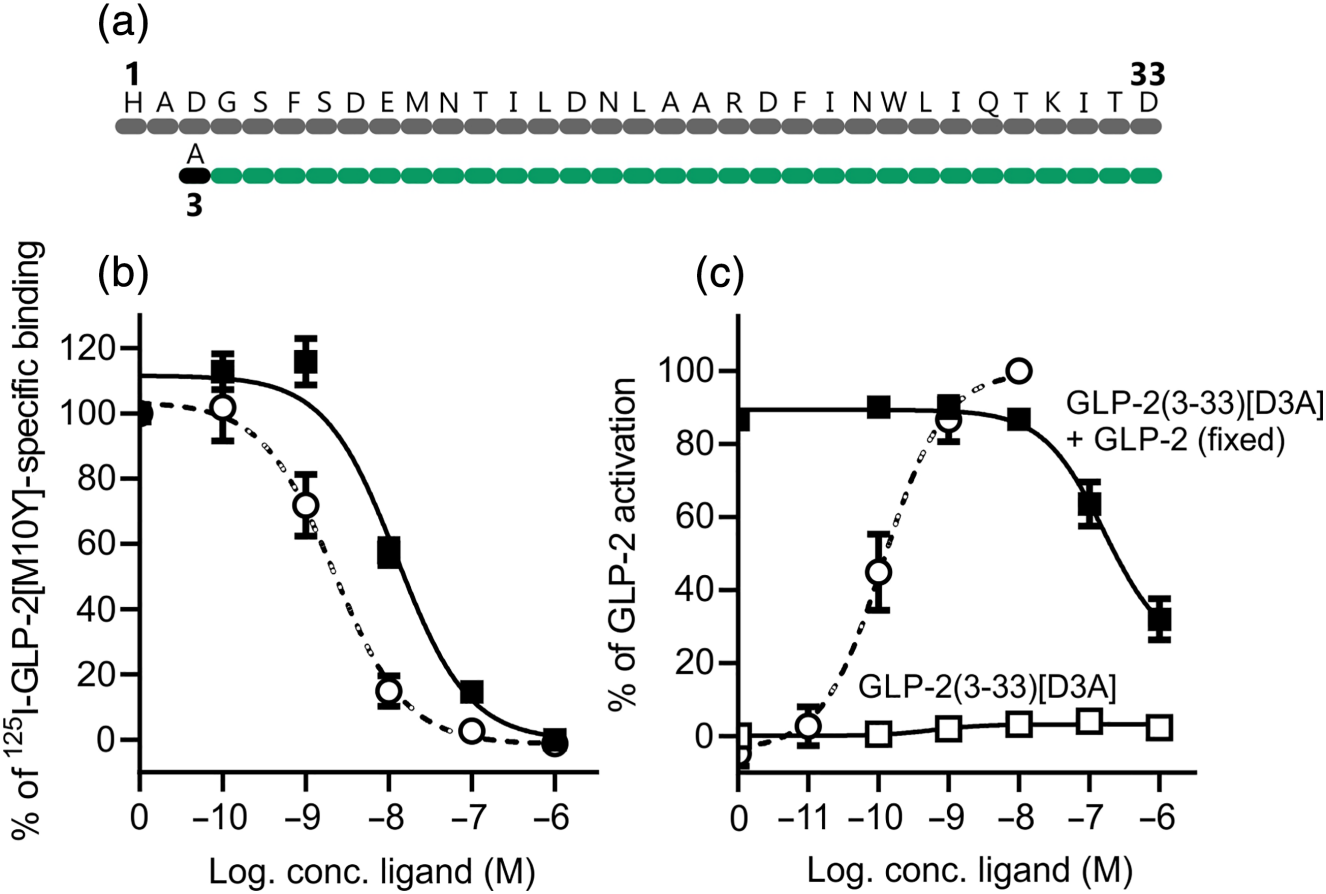
cAMP activation and inhibition profiles of GLP‐2(3‐33)[D3A] on the human GLP‐2R. (a) Schematic overview of GLP‐2(3‐33)[D3A]. The black dot indicates where the amino acid has been substituted. COS‐7 cells were transiently transfected with the human GLP‐2R and assessed for (b) competition with [^125^I]‐GLP‐2(1‐33)[M10Y] or (c) cAMP activation or inhibition with GLP‐2(3‐33)[D3A]. The dashed line represents human GLP‐2. Data are shown as mean ± SEM, from *n* = 3 independent experiments carried out in duplicate

As described above, the naturally occurring DPP‐4 cleavage product of GLP‐2, GLP‐2(3‐33), is a partial agonist with antagonistic properties as previously observed (Thulesen et al., 2002; Yamazaki et al., 2013). In attempts to improve the antagonistic properties and limit the possibility of GLP‐2R activation, we substituted the Asp at position 3 with Ala, creating GLP‐2(3‐33)[D3A] (Figure 3a). This peptide variant had a K_i_ value of 13 nM, thus similar to that of GLP‐2(3‐33) (Figure 3b), but the amino acid substitution completely eliminated the agonist activity observed for GLP‐2(3‐33). Furthermore, GLP‐2(3‐33)[D3A] inhibited the GLP‐2‐mediated activity at the GLP‐2R with an IC_50_ value of 162 nM, similar to that of GLP‐2(3‐33) (Figure 3c).

### 3.4 GLP‐2(2‐33) and GLP‐2(3‐33)[D3A] are competitive antagonists of the GLP‐2R

**FIGURE 4:**
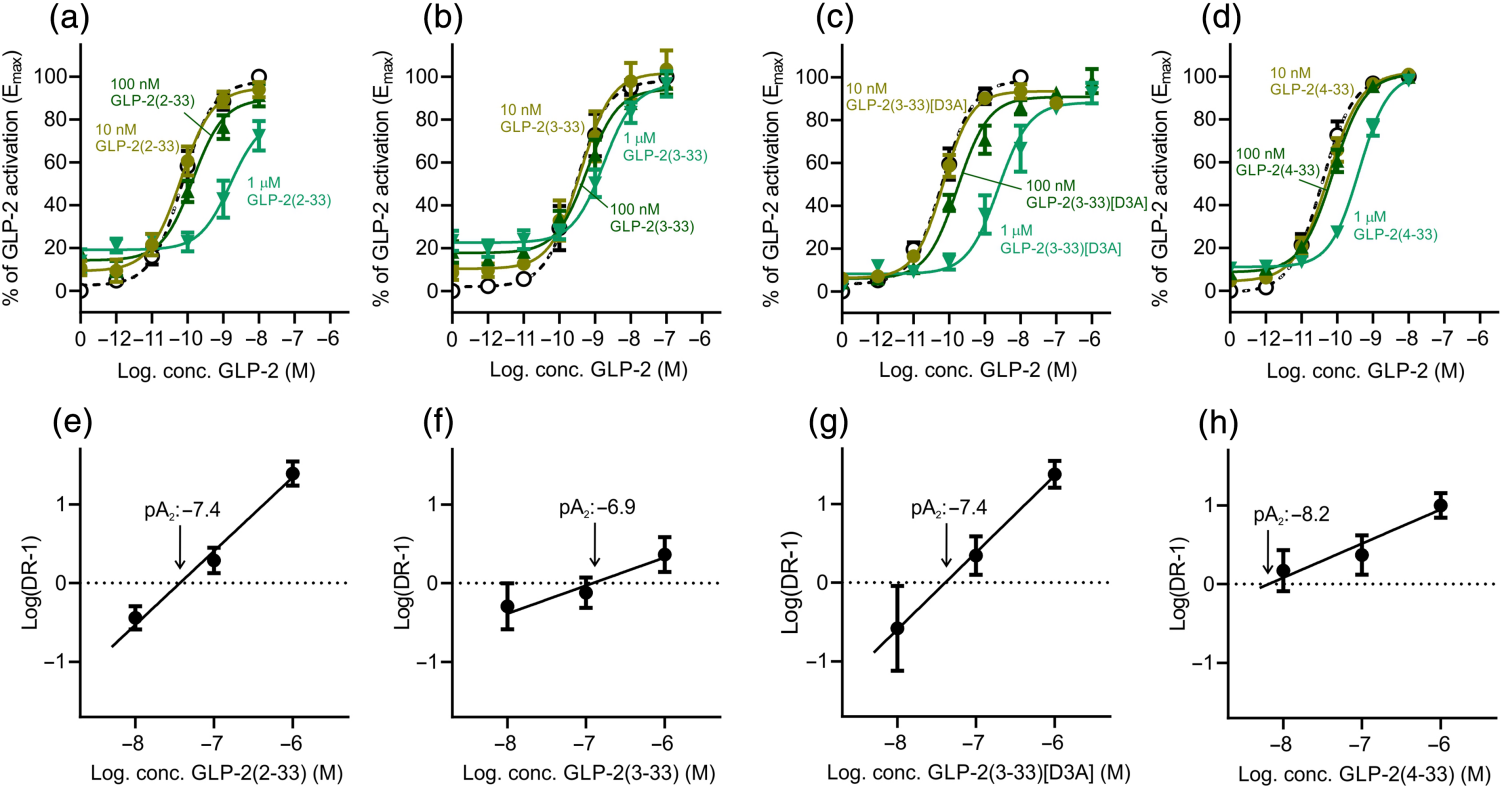
Schild plots of GLP‐2(2‐33), GLP‐2(3‐33), GLP‐2(3‐33)[D3A] and GLP‐2(4‐33) on the human GLP‐2R. COS‐7 cells were transiently transfected with the human GLP‐2R and assessed for cAMP accumulation upon ligand stimulation with GLP‐2 in the absence or presence of increasing concentrations (a) GLP‐2(2‐33), (b) GLP‐2(3‐33), (c) GLP‐2(3‐33)[D3A] and (d) GLP‐2(4‐33). The corresponding Schild plots of (e) GLP‐2(2‐33), (f) GLP‐2(3‐33), (g) GLP‐2(3‐33)[D3A] and (h) GLP‐2(4‐33) indicating their respective pA_2_ value. Data are shown as mean ± SEM, from *n* = 5 independent experiments carried out in duplicate.

To further examine the pharmacodynamic properties of the most potent antagonists (GLP‐2(2‐33), GLP‐2(3‐33), GLP‐2(3‐33)[D3A] and GLP‐2(4‐33)), we studied whether they were competitive antagonists. We used a Schild plot approach, where we examined their ability to right‐shift the cAMP activation curve of native GLP‐2 (Figure 4a–d). For GLP‐2(2‐33), GLP‐(3‐33) and GLP‐2(4‐33), all having intrinsic activity, the Schild plot analysis was based on an equieffective DR (the concentrations at 60% of E_max_). For GLP‐2(3‐33)[D3A], with no intrinsic activity, the Schild plot analysis was conducted based on the shift in the EC_50_ values for GLP‐2 with and without GLP‐2(3‐33)[D3A] as previously described (Sparre‐Ulrich et al., 2017). For GLP‐2(2‐33), the slope was found to be 0.94 ± 0.12 and pA_2_ −7.4 (K_i_ 40 nM) (Figure 4e), for GLP‐2(3‐33), the slope was 0.36 ± 0.17 and pA_2_ −6.9 (K_i_ 126 nM) (Figure 4f), for GLP‐2(3‐33)[D3A], the slope was 0.98 ± 0.24 and pA_2_ −7.4 (K_i_ 40 nM) (Figure 4g) and for GLP‐2(4‐33), the slope was 0.43 ± 0.15 and pA_2_ −8.2 (K_i_ 6.3 nM) (Figure 4h). Based on these results, only GLP‐2(2‐33) and GL‐2(3‐33)[D3A] were competitive antagonists because the slopes of their Schild plots were not significantly different from 1 (unpaired *t*‐test with Welch's correction).

### 3.5 N‐terminal truncation of GLP‐2 leads to increasing loss of selectivity for the human GLP‐2R

**FIGURE 5:**
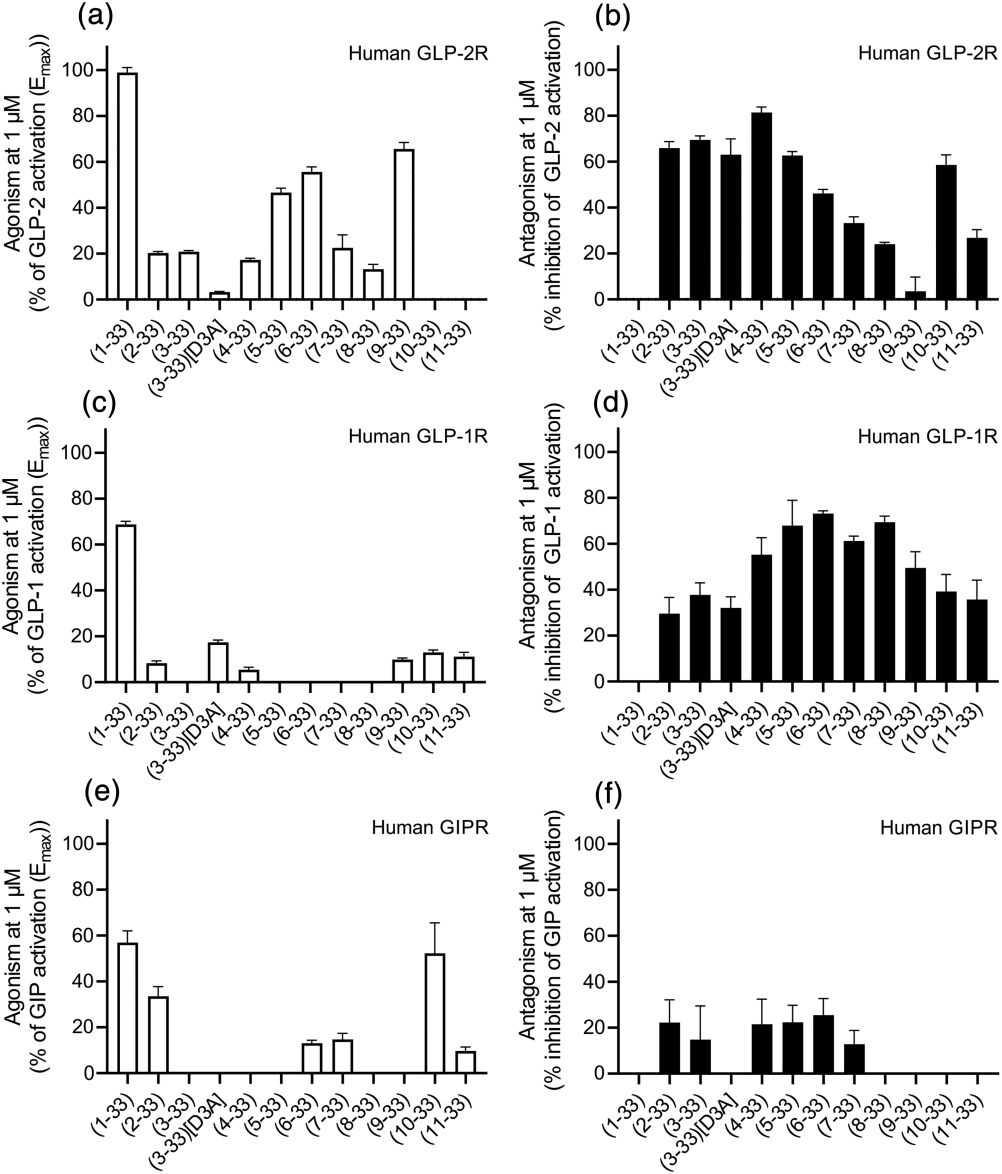
Selectivity test of the N‐terminally truncated GLP‐2 variants on human GLP‐1R and human GIPR. COS‐7 cells were transiently transfected with either human GLP‐2R, human GLP‐1R or human GIPR, and the agonistic and antagonistic profiles of the N‐terminally truncated GLP‐2 variants were examined in cAMP accumulation. (a) Agonistic and (b) antagonistic activities of the N‐terminally truncated GLP‐2 variants on human GLP‐2R, (c) agonistic and (d) antagonistic activities of the N‐terminally truncated GLP‐2 variants on the human GLP‐1R and (e) agonistic and (f) antagonistic activites of the N‐terminally truncated GLP‐2 variants on the human GIPR. Data are shown as mean ± SEM, from *n* = 3 independent experiments carried out in duplicate

Given the similar binding pattern of endogenous agonists in class B1 G protein‐coupled receptors (GPCRs), and the recently described activity of GLP‐2 at the GIPR (Skov‐Jeppesen et al., 2019) and the GLP‐1R (Gadgaard et al., 2021), we examined the effects on selectivity towards these receptors by removal of residues of the N‐terminus (Figure 5). For the human GLP‐1R, no major agonistic activity was observed for any of the N‐terminal truncated GLP‐2 variants (Figure 5c compared with Figure 5a). However, when testing their ability to inhibit the GLP‐1R, all truncations had some antagonistic properties (>20% inhibition) at the highest tested concentration of 1 μM (Figure 5d). Interestingly, the antagonistic properties of the N‐terminally truncated GLP‐2 peptides at the GLP‐1R were weaker for the peptides lacking the first two N‐terminal amino acids (GLP‐2(2‐33), GLP‐2(3‐33)), which inhibited GLP‐1‐mediated response by only 30%–38%. Strongest antagonism was observed for the middle truncations (GLP‐2(6‐ to 8‐33)) ranging between 61% and 73% inhibition. This was a trend opposite to that observed with the GLP‐2R where the most potent antagonism was observed for the first N‐terminal truncations and less for the middle N‐terminal truncations (Figure 5b). With respect to the human GIPR, some agonist activity was observed for GLP‐2(2‐33) and GLP‐2(10‐33) at 1 μM of the peptides (Figure 5e), whereas none of the truncations were able to inhibit the GIPR noticeably(Figure 5f).

### 3.6 Lipidation of N‐terminally truncated GLP‐2 peptides improves the antagonistic profile but also improves GLP‐1R interaction

**FIGURE 6:**
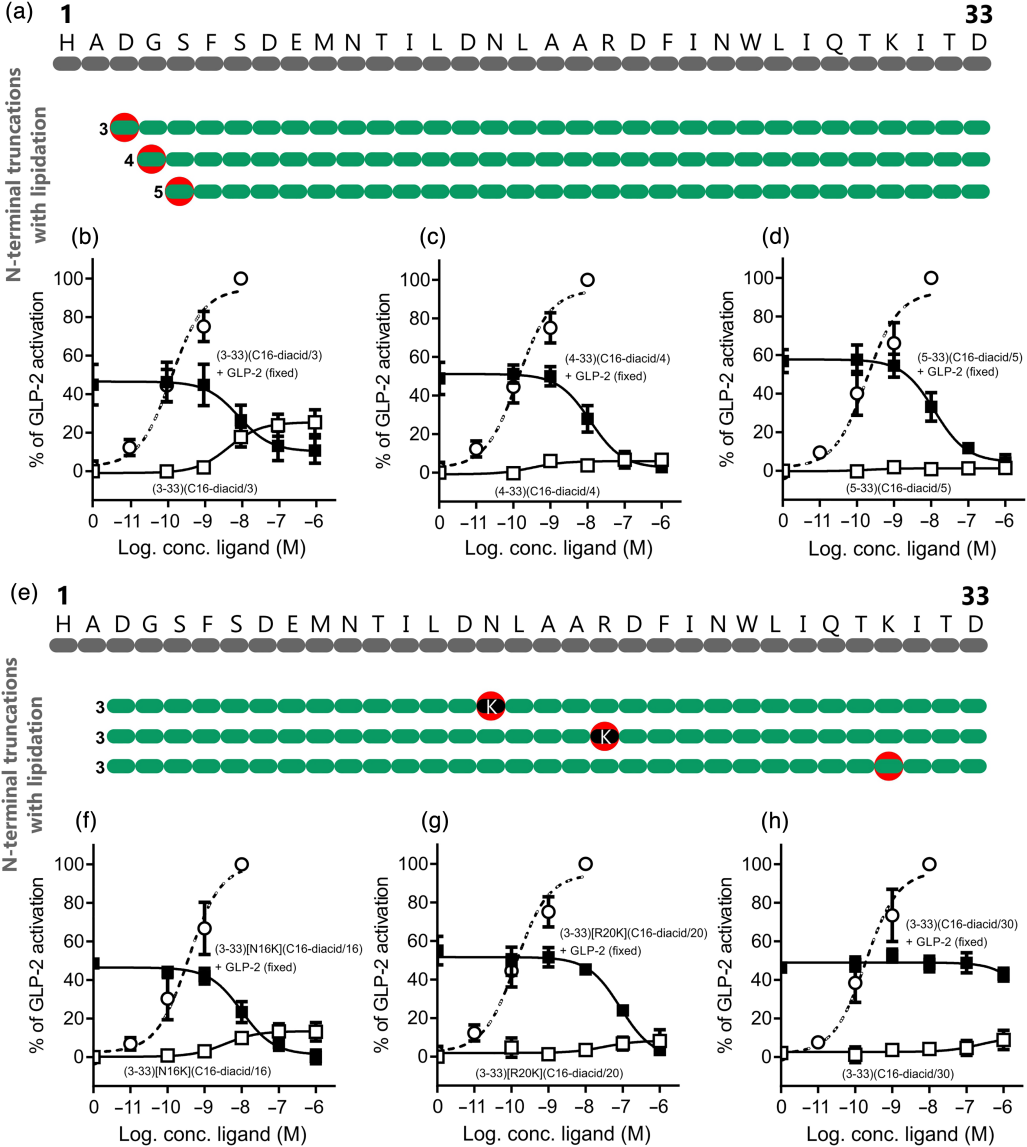
cAMP activation and inhibition profiles of N‐terminally truncated and lipidated GLP‐2 variants on the human GLP‐2R. (a) Schematic overview of the N‐terminally truncated lipidated GLP‐2 variants. The red circles indicate where a 16‐carbon fatty diacid chain has been attached. COS‐7 cells were transiently transfected with human GLP‐2R and assessed for either cAMP activation or inhibition with (b) GLP‐2(3‐33)(C16‐diacid/3), (c) GLP‐2(4‐33)(C16‐diacid/4) or (d) GLP‐2(5‐33)(C16‐diacid/5). (e) Schematic overview of the N‐terminally truncated amino acid modified lipidated GLP‐2 variants. The red circles indicate where a 16‐carbon fatty diacid chain has been attached, and the black dots indicate where an amino acid has been substituted. COS‐7 cells were transiently transfected with the human GLP‐2R and assessed for either cAMP activation or inhibition with (f) GLP‐2(3‐33)[N16K](C16‐diacid/16), (g) GLP‐2(3‐33)[R20K](C16‐diacid/20) or (h) GLP‐2(3‐33)(C16‐diacid/30). The dashed line represents human GLP‐2. Data are shown as mean ± SEM, from *n* = 3 independent experiments carried out in duplicate.

The conclusion from the activity probing was that the best antagonistic profile on the human GLP‐2R was observed for the first N‐terminal truncated GLP‐2 peptides (Figures 2 and 5b). To make the GLP‐2R antagonists more suitable for in vivo studies and future clinical use, we wanted to increase their half‐life by site‐specific lipidation. First, we lipidated GLP‐2(3‐33), GLP‐2(4‐33) and GLP‐2(5‐33) by attaching a lipid chain of 16 carbon atoms (hexadecanedioic acid abbreviated to C16‐diacid in this paper) directly to their N‐terminus (Figure 6a). To attach a fatty acid to the middle of the peptide, we substituted both of the amino acids at positions 16 and 20 in GLP‐2(3‐33) with Lys and attached a C16‐diacid to their side chains (Figure 6e). Also, position 30 in GLP‐2(3‐33) was lipidated (Figure 6e). The N‐terminal lipidation of GLP‐2(3‐33), GLP‐2(4‐33) and GLP‐2(5‐33) resulted in very potent antagonists of the GLP‐2R with IC_50_ values of 7.9, 11.7 and 12.3 nM, respectively, where only the GLP‐2(3‐33) lipidated variant had remaining agonism (E_max_ 25%) (Figure 6b–d). Compared with their corresponding non‐lipidated versions, they were 26‐, 7‐ and 16‐fold more potent, respectively. When comparing the Schild plots for the N‐terminal lipidation of GLP‐2(3‐33) and GLP‐2(4‐33) with their non‐lipidated versions, they were also better (Figure 4fh/ and Figure S1b/f). Likewise, lipidation in the middle of the peptide at position 16 (GLP‐2(3‐33)[N16K](C16‐diacid/16)) resulted in potent antagonism (IC_50_ 10 nM, Figure 6f), corresponding to a 20‐fold improvement compared with GLP‐2(3‐33) and higher affinity, K_i_ 40 nM (Figure S1h) compared with K_i_ 126 nM (Figure 4f). In contrast, lipidation at position 20 (GLP‐2(3‐33)[R20K](C16‐diacid/20)) resulted in weaker antagonism (IC_50_ 85 nM and K_i_ 398 nM) (Figure 6g and Figure S1j), and the compound with lipidation at position 30 (GLP‐2(3‐33)(C16‐diacid/30)) was not able to antagonize GLP‐2‐induced activity of the GLP‐2R (Figure 6h).

**FIGURE 7:**
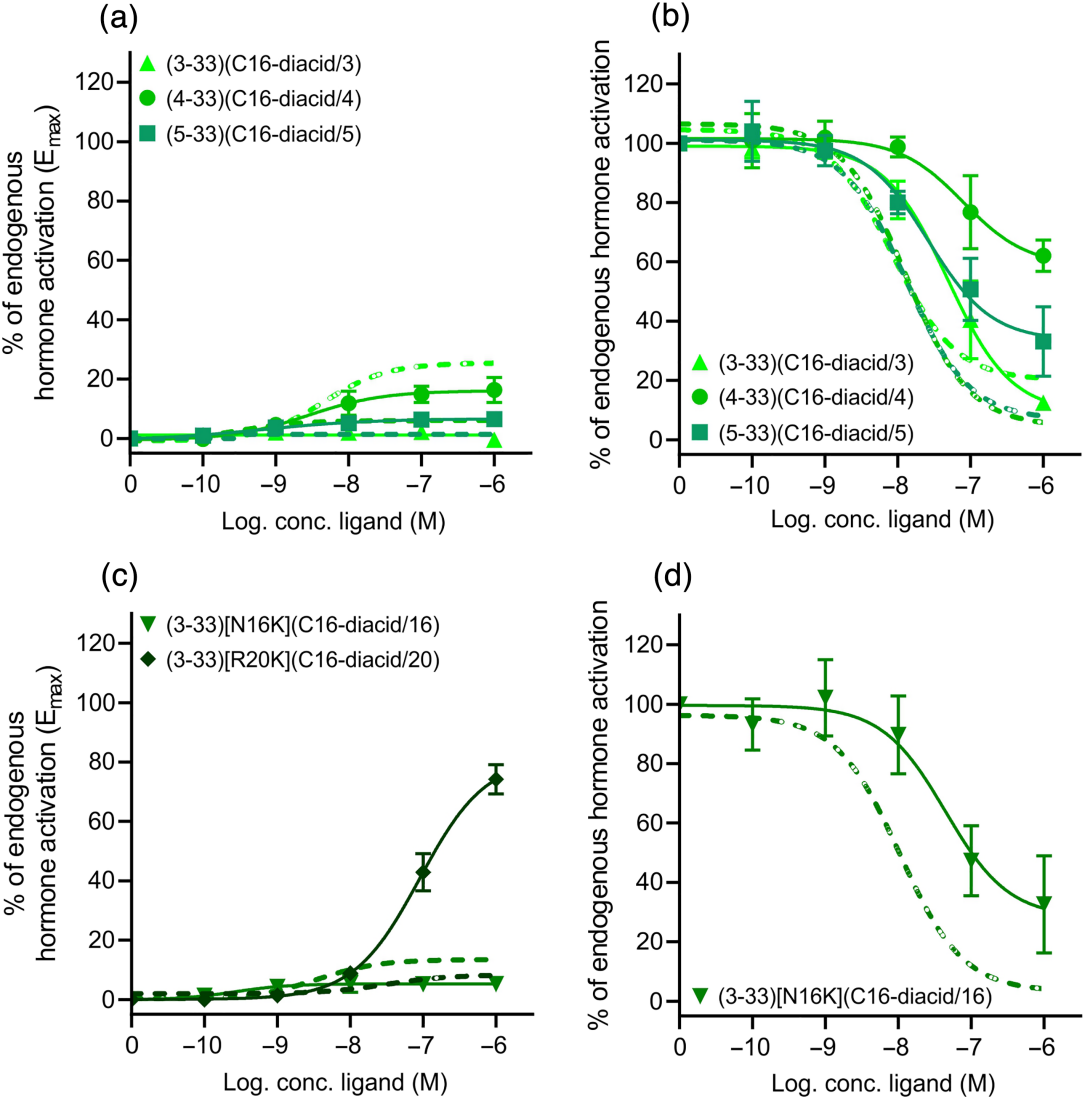
Selectivity test of the N‐terminally truncated and lipidated GLP‐2 variants on the human GLP‐1R. COS‐7 cells were transiently transfected with the human GLP‐2R or human GLP‐1R and assessed for either cAMP activation (a) or inhibition (b) with GLP‐2(3‐33)(C16‐diacid/3), GLP‐2(4‐33)(C16‐diacid/4) and GLP‐2(5‐33)(C16‐diacid/5) or cAMP activation (c) or inhibition (d) with GLP‐2(3‐33)[N16K](C16‐diacid/16) and GLP‐2(3‐33)[R20K](C16‐diacid/20). The full lines represent the human GLP‐1R, and the dashed lines the human GLP‐2R. Data are shown as mean ± SEM, from *n* = 3 independent experiments carried out in duplicate

For the lipidated GLP‐2R antagonists, we also tested the selectivity and focused on only the human GLP‐1R as the N‐terminal truncations were most promiscuous for this receptor (Figure 5). Again, very little intrinsic activity was observed for the peptides at the GLP‐1R at 1 μM stimulation except for GLP‐2(4‐33)(C16‐diacid/4) and GLP‐2(3‐33)[R20K](C16‐diacid/20), which activated the GLP‐1R with efficacies of 16% and 82%, and potencies of 2.5 and 100 nM, respectively (Figure 7a,c, respectively). The antagonistic profile of the N‐terminally lipidated GLP‐2R antagonists varied a lot on the human GLP‐1R (Figure 7b). As such, lipidation at position 3 (GLP‐2(3‐33)(C16‐diacid/3)) gave GLP‐1R inhibition by 91%, at position 4 (GLP‐2(4‐33)(C16‐diacid/4)) 41% and at positon 5 (GLP‐2(5‐33)(C16‐diacid/5)) 67% inhibition compared with 87%, 95% and 93% on the human GLP‐2R, respectively (Figure 7b). The potencies were 50, 79 and 40 nM for GLP‐2(3‐33)(C16‐diacid/3), GLP‐2(4‐33)(C16‐diacid/4) and GLP‐2(5‐33)(C16‐diacid/5), respectively, that is, also less potent than on the human GLP‐2R (~2–5 fold) (Figure 7b). Lipidation in the middle of the peptide did not change the selectivity pattern as GLP‐2(3‐33)[N16K](C16‐diacid/16) was still able to inhibit the GLP‐1R with 72% at 1 μM stimulation compared with 97% at the GLP‐2R (Figure 7d) with a potency of 40 nM (4‐fold less potent than on the human GLP‐2R).

## 4 DISCUSSION

In this molecular pharmacological study of N‐terminal truncations of human GLP‐2, we evaluated GLP‐2(2‐ to 11‐33) as antagonists of the human GLP‐2R. A gradual loss in GLP‐2R affinity was observed with reduced N‐terminal GLP‐2 peptide length. GLP‐2(2‐ to 4‐33) had very intrinsic little activity, whereas GLP‐2(5‐ to 6‐33) and GLP‐2(9‐33) were low potent partial agonists at the GLP‐2R, where increasing efficacy appeared when more of the N‐terminus was removed. GLP(2‐ to 5‐33) were able to antagonize the human GLP‐2R, but only GLP‐2(2‐33) and GLP‐2(3‐33)[D3A] were competitive antagonists. The N‐terminally truncated GLP‐2 peptides were not particularly selective as most of them were also able to antagonize the human GLP‐1R, and the selectivity decreased when more of the N‐terminus was removed. Lipidation of the N‐terminally truncated GLP‐2 peptides improved the antagonistic properties at the human GLP‐2R, but not the selectivity for this receptor.

N‐terminal truncations of the GLP‐2 peptide have previously been characterized, only very sparsely (Thulesen et al., 2002; Yamazaki et al., 2013) and a systematic approach as carried out here has not been presented before. The first N‐terminally truncated GLP‐2 peptide studied was GLP‐2(3‐33), the DPP‐4 product of native GLP‐2, which showed weak partial agonism (efficacy of 15% and potency >150‐fold right‐shifted compared with native GLP‐2) in cAMP accumulation experiments using BHK cells stably expressing the human GLP‐2R (Thulesen et al., 2002). The activity of GLP‐2(3‐33) wassubsequently tested in HEK293 cells stably expressing the human GLP‐2R. Here GLP‐2(3‐33) again showed partial agonism, in these cells with an efficacy of 58% and a potency >300‐fold right‐shifted compared with native GLP‐2 (Yamazaki et al., 2013). We likewise observed that GLP‐2(3‐33) was a partial agonist with a similar activation profile, as described in the BHK cells (Thulesen et al., 2002) (Figure 2b and Table 1). Despite this low partial agonism, it still had antagonistic properties in vivo as shown by its inhibition of the gut proliferative actions of GLP‐2 in mice as estimated from crypt cell proliferation and apoptosis (Baldassano et al., 2013; Thulesen et al., 2002). Furthermore, the activation profiles of GLP‐2(6‐33) and GLP‐2(11‐33) have been described (Yamazaki et al., 2013). For GLP‐2(6‐33), a high intrinsic activity was also observed (efficacy of 68% and potency of 25 nM), which is very similar to our observations (Figure 2e and Table 1). Thus, it is previously presented that N‐terminal truncation of GLP‐2 can result in partial agonists. In contrast, the activation profile of GLP‐2(11‐33) in our studies did not reflect what had previously been observed. Of all of the N‐terminally truncated GLP‐2 peptides, GLP‐2(11‐33) is the one with the poorest affinity (~400‐fold impaired compared with human GLP‐2) (Figure 1b/c, Table 1) and had neither agonistic nor antagonistic properties (Figure 2j, Table 1). This reflects that reducing the N‐terminus with 10 amino acids results in a peptide that is no longer able to bind to the receptor and for all of the N‐terminal truncations we observe a decrease in affinity with reduced N‐terminal GLP‐2 peptide length (Figure 1b). This correlates well with the notion within the class B1 GPCR ligands, with the α‐helix (spanning from amino acid 4 to 29 in GLP‐2) (Figure 1a) being the affinity generating part, and the N‐terminus providing the efficacy (Hoare, 2005; Schwartz & Frimurer, 2017). However, opposed to this, GLP‐2(11‐33) has previously been reported to retain the binding activities of 88% at 1 μM and 100% at 10 μM compared with native GLP‐2, and it was also shown to have an agonistic profile with an efficacy of 11% compared with that of GLP‐2 (Yamazaki et al., 2013). From these studies, it was concluded that GLP‐2(11‐33) is a potent orthosteric GLP‐2R antagonist as it decreased the agonistic activity of an ago‐allosteric modulator on the rat GLP‐2R (Yamazaki et al., 2013). However, it has to be taken into consideration that different experimental methods, cell types and receptor species were applied in the studies, which may explain the observed discrepancies.

A similar approach, as we applied here in the search for GLP‐2R antagonists, has been applied previously for the GIP system. Here sequential N‐terminal truncations of the first eight amino acids of GIP(1–30)NH_2_ identified GIP(3–30)NH_2_ and GIP(5–30)NH_2_ as potent, competitive GIPR antagonists with no intrinsic activity (Hansen et al., 2016). For these truncated GIP peptides, the affinity also decreased with truncation length from GIP(5–30)NH_2_ and upwards, and the best antagonists were found among the first four N‐terminal truncations. As GIP(3–30)NH_2_ is the naturally occurring DPP‐4 degradation product of GIP(1–30)NH_2_ (like GLP‐2(3‐33) is for GLP‐2(1‐33)), this antagonist was studied further and found to be selective for only the GIPR, among a large group of tested class B1 GPCRs (Gabe et al., 2018; Gasbjerg et al., 2017). In contrast, N‐terminally truncated GLP‐2 peptides did not result in selectivity for the GLP2R as they all inhibit the GLP‐1R, and the selectivity was observed to decrease with reduced peptide length (Figure 5d). It is known that GLP‐2 is an agonist with low potency, on the GLP‐1R (Gadgaard et al., 2021) and that the N‐terminal truncations in GLP‐2 do not impair its ability to interact with the GLP‐1R. Selectivity is extremely important if the antagonist is to be used to study GLP‐2 physiology further as any effects otherwise cannot be ascribed the GLP‐2 system. Selectivity, however, presents a common challenge within the class B1 GPCRs, as the endogenous ligands for these receptors are closely related (Couvineau & Laburthe, 2012) and the hormones sometimes activate more than one receptor. This has for instance been observed for glucagon and oxyntomodulin that act as agonists on both the glucagon receptor and the GLP‐1R (Jorgensen et al., 2007; Svendsen et al., 2018) and GLP‐2 that is an agonist with low potency at the GIPR  (Skov‐Jeppesen et al., 2019). Similarity in structure and receptor activation pattern enhances the probability of co‐targeting the receptors, a finding that has already been exploited extensively within the class B1 system (Finan et al., 2013, 2014; Willard et al., 2020).

Whether GLP‐2R antagonists have therapeutic potential is still uncertain. Treatment with teduglutide in patients with SBS appears to reduce gastric emptying and stomal output, to increase intestinal energy absorption and to promote weight gain (Bremholm et al., 2009; Drucker & Yusta, 2014; Jeppesen et al., 2001). Therefore, blockade of the GLP‐2R signal could theoretically reduce general nutrient absorption pointing towards a potential role for GLP‐2R antagonists in the treatment of obesity. This is supported by observations that GLP‐2 rapidly augments the uptake of lipids and enhances triglyceride‐rich‐chylomicron secretion from the gut mucosa in mice (Hsieh et al., 2009). Likewise, acute administration of GLP‐2 increases plasma triglyceride and free‐fatty‐acid levels in healthy human subjects during a meal test (Meier et al., 2006). GLP‐2 has, however, also been shown to decrease food intake when administered intracerebroventricularly in mice (Guan et al., 2012). In contrast, no alterations in body weight or fat mass appeared after 3 weeks of treatment with GLP‐2(3‐33) administration subcutaneously in rats (Baldassano et al., 2019). Here, species differences have to be taken into consideration. Moreover the GLP‐2 actions ascribed to studies using GLP‐2(3‐33) may also be reconsidered because it also antagonizes the GLP‐1Rr (Figure 5d). Thus, further studies are needed to examine the role of the GLP‐2 systems in human physiology to fully understand its therapeutic potential. Our study shows that the N‐terminus of GLP‐2 is essential for GLP‐2R activity and that the selectivity towards the GLP‐2R decreases with reduced N‐terminal GLP‐2 peptide length. This provides valuable information for further development of selective GLP‐2‐based tool compounds.

## AUTHOR CONTRIBUTIONS

Designing research studies: MBNG, JJH and MMR. Conducting experiments: MBNG, SG and PL. Analysing data: MBNG and MMR. Writing the manuscript: MBNG, LSG, JJH and MMR. Review and editing of manuscript: MBNG, LSG, SG, PL, JJH and MMR.

## CONFLICT OF INTERESTS

The authors declare that the research was conducted in the absence of any commercial or financial relationships that could be construed as a potential conflict of interest. MBNG, LSG, JJH and MMR are co‐founders of Antag Therapeutics ApS. MMR and JJH are also co‐founders of Bainan Biotech ApS.

## DECLARATION OF TRANSPARENCY AND SCIENTIFIC RIGOUR

This Declaration acknowledges that this paper adheres to the principles for transparent reporting and scientific rigour of preclinical research as stated in the *BJP* guidelines for Design and Analysis, and as recommended by the funding agencies, publishers and other organizations engaged with supporting research.

## Supporting information


**Figure S1.** Schild plots of N‐terminally truncated lipidated GLP‐2 variants on the human GLP‐2R. COS‐7 cells were transiently transfected with the human GLP‐2R and assessed for cAMP accumulation upon ligand stimulation of GLP‐2 in the absence or presence of increasing concentrations of the N‐terminally truncated lipidated GLP‐2 variants and their corresponding Schild plots were drawn to obtain pA_2_ values for (A/B) GLP‐2(3‐33)(C16‐diacid/3), (C/D) GLP‐2(4‐33)(C16‐diacid/4), (E/F) GLP‐2(5‐33)(C16‐diacid/5), (G/H) GLP‐2(3‐33)[N16K](C16‐diacid/16) and (I/J) GLP‐2(3‐33)[R20K](C16‐diacid/20). The dashed line represents human GLP‐2 in the absence of any N‐terminally truncated lipidated GLP‐2 variant. Data are shown as mean ± SEM, n = 5 independent experiments carried out in duplicate.

## Data Availability

The data that support the findings of this study are available from the corresponding author upon reasonable request.
