# Supplementary figures and images for: N‐terminal alterations turn the gut hormone GLP‐2 into an antagonist with gradual loss of GLP‐2 receptor selectivity towards more GLP‐1 receptor interaction
Source: Br J Pharmacol. 2022 Jun 8;179(18):4473–85. doi: 10.1111/bph.15866 (PMC9541843; doi:10.1111/bph.15866)

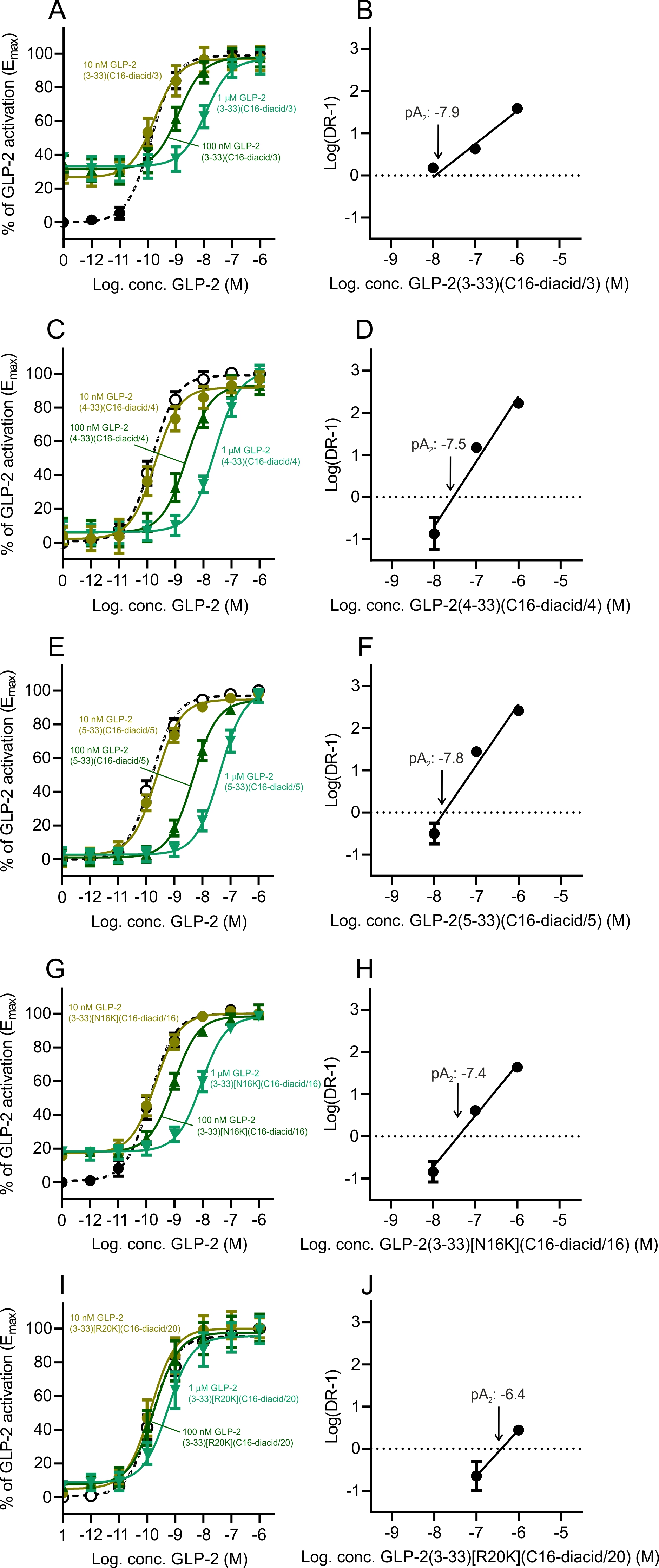

Supplement: Supplementary file 1 — Figure S1. Schild plots of N‐terminally truncated lipidated GLP‐2 variants on the human GLP‐2R. COS‐7 cells were transiently transfected with the human GLP‐2R and assessed for cAMP accumulation upon ligand stimulation of GLP‐2 in the absence or presence of increasing concentrations of the N‐terminally truncated lipidated GLP‐2 variants and their corresponding Schild plots were drawn to obtain pA2 values for (A/B) GLP‐2(3‐33)(C16‐diacid/3), (C/D) GLP‐2(4‐33)(C16‐diacid/4), (E/F) GLP‐2(5‐33)(C16‐diacid/5), (G/H) GLP‐2(3‐33)[N16K](C16‐diacid/16) and (I/J) GLP‐2(3‐33)[R20K](C16‐diacid/20). The dashed line represents human GLP‐2 in the absence of any N‐terminally truncated lipidated GLP‐2 variant. Data are shown as mean ± SEM, n = 5 independent experiments carried out in duplicate. [file BPH-179-4473-s001.jpg]
